# Human embryoid bodies as a 3D tissue model of the extracellular matrix and α-dystroglycanopathies

**DOI:** 10.1242/dmm.042986

**Published:** 2020-06-26

**Authors:** Alec R. Nickolls, Michelle M. Lee, Kristen Zukosky, Barbara S. Mallon, Carsten G. Bönnemann

**Affiliations:** 1 National Institute of Neurological Disorders and Stroke, National Institutes of Health, Bethesda, MD 20892, USA; 2 Department of Neuroscience, Brown University, Providence, RI 02912, USA

**Keywords:** Dystroglycan, Extracellular matrix, Muscular dystrophy, Stem cells

## Abstract

The basal lamina is a specialized sheet of dense extracellular matrix (ECM) linked to the plasma membrane of specific cell types in their tissue context, which serves as a structural scaffold for organ genesis and maintenance. Disruption of the basal lamina and its functions is central to many disease processes, including cancer metastasis, kidney disease, eye disease, muscular dystrophies and specific types of brain malformation. The latter three pathologies occur in the α-dystroglycanopathies, which are caused by dysfunction of the ECM receptor α-dystroglycan. However, opportunities to study the basal lamina in various human disease tissues are restricted owing to its limited accessibility. Here, we report the generation of embryoid bodies from human induced pluripotent stem cells that model the basal lamina. Embryoid bodies cultured via this protocol mimic pre-gastrulation embryonic development, consisting of an epithelial core surrounded by a basal lamina and a peripheral layer of ECM-secreting endoderm. In α-dystroglycanopathy patient embryoid bodies, electron and fluorescence microscopy reveal ultrastructural basal lamina defects and reduced ECM accumulation. By starting from patient-derived cells, these results establish a method for the *in vitro* synthesis of patient-specific basal lamina and recapitulate disease-relevant ECM defects seen in the α-dystroglycanopathies. Finally, we apply this system to evaluate an experimental ribitol supplement therapy on genetically diverse α-dystroglycanopathy patient samples.

This article has an associated First Person interview with the first author of the paper.

## Abstract (precis)

**Summary:** Human stem cell culture methods enable the generation of extracellular matrix-containing tissue to study patient-specific pathology and experimental treatment of the α-dystroglycanopathies.

## INTRODUCTION

Metazoan life relies on tissue compartmentalization to form ordered, discrete organs. This is partly accomplished by an extracellular matrix (ECM) barrier called the basal lamina, which ensheaths epithelial, endothelial, adipose, muscle and nervous tissue (Morrissey and Sherwood, 2015). The main components comprising the basal lamina are laminin isoforms, perlecan, nidogen and collagen type IV, forming a complex lattice anchored to cell surface receptors (Yurchenco, 2011). This cell-ensheathing basal lamina is generally inter-connected on its acellular matrix side to a ‘lamina reticularis’ composed of fibrillar collagens, microfibrils and proteoglycans. Together, they form a multilayered basement membrane with tissue-specific mechanical properties (Sanes, 1982; Yurchenco, 2011). The terms ‘basal lamina’ and ‘basement membrane’ are sometimes used interchangeably. However, here they will refer to distinct structures, with the basal lamina being the dense, cell-attached component of the basement membrane.

The basal lamina is an essential structural component for organ genesis and maintenance. Its perturbation is linked to many human clinical conditions including metastatic cancer, nephropathy, lissencephaly and muscular dystrophy. One mechanistic group of basal lamina-related diseases pertains to the dysfunction of the cell membrane ECM receptors integrin and dystroglycan (Godfrey et al., 2011; Winograd-Katz et al., 2014). These receptors mediate cell attachment to the basal lamina and, in turn, they influence the arrangement of the basal lamina itself (Li et al., 2002, 2017; Moore et al., 2002; Han et al., 2009).

Dystroglycan is composed of two protein subunits both encoded by the *DAG1* gene: α-dystroglycan (αDG) and β-dystroglycan (βDG). αDG is located at the cell surface and directly binds to the ECM, whereas βDG is a transmembrane protein that links αDG to intracellular structural and signaling proteins (Ibraghimov-Beskrovnaya et al., 1992; Holt et al., 2000). There is an expanding literature on the spectrum of disorders caused by αDG receptor dysfunction, collectively termed the α-dystroglycanopathies. A hallmark of severe α-dystroglycanopathies is rupture or detachment of the basal lamina that encases the brain and muscle fibers during development and structural maintenance (Ishii et al., 1997; Devisme et al., 2012). This specific combination of basal lamina abnormalities is associated with a range of developmental nervous system malformations and progressive skeletal muscle degeneration that can ultimately be fatal.

The biochemical basis of the α-dystroglycanopathies is a reduction in a highly specific form of O-linked glycosylation on αDG. This leads to a ‘hypoglycosylation’ of the final αDG glycoepitope – a post-translational structure referred to as the matriglycan (Yoshida-Moriguchi and Campbell, 2015). Normal matriglycans on αDG confer binding activity to the ECM molecules laminin, perlecan and agrin (Briggs et al., 2016). Hypoglycosylated matriglycans have limited ECM binding capacity, which is thought to destabilize the basal lamina in muscle and brain tissue, and represents a common disease pathway in the α-dystroglycanopathies (Michele et al., 2002; Moore et al., 2002). The 17 genes that are known to be mutated in the α-dystroglycanopathies all affect the formation of matriglycans. These genes encode various specific glycosyltransferases as well as enzymes preparing specific sugars to be incorporated into the matriglycan structure, and very few α-dystroglycanopathy cases involve mutations in the *DAG1* gene itself (Yoshida-Moriguchi and Campbell, 2015). Based on this knowledge, a large proportion of α-dystroglycanopathy cases can now be clarified genetically (Clement et al., 2012; Graziano et al., 2015).

Understanding the mechanisms of pathogenesis and developing rational therapies for the α-dystroglycanopathies remains a challenge, in part because of its phenotypic and genetic heterogeneity. A large collection of α-dystroglycanopathy animal models recapitulate many aspects of the clinical spectrum (Nickolls and Bönnemann, 2018). However, such approaches fall short of modeling the genetic diversity of human patients for assessing disease phenotypes and drug responses.

To study patient-specific basal lamina in a model system, we developed a protocol to generate ECM-containing spheroids from human induced pluripotent stem cells (hiPSCs), which we refer to as embryoid bodies. hiPSC-derived embryoid bodies produce their own basal lamina and represent a simplified 3D system to investigate human ECM and its receptors in diverse genetic contexts. As a proof of concept, we applied this method to produce embryoid bodies from a variety of α-dystroglycanopathy patients. We observed subtle basal lamina defects that correlated with disease severity and corroborate findings in mouse models. Lastly, we evaluated patient hiPSCs and embryoid bodies treated with the sugar alcohol ribitol, a recently proposed therapeutic for the α-dystroglycanopathies. By correlating a patient’s genotype and drug response, this approach allows for pre-clinical prediction of therapeutic efficacy in specific individuals.

## RESULTS

### Human embryoid bodies mimic pre-gastrulation development

**Fig. 1.**
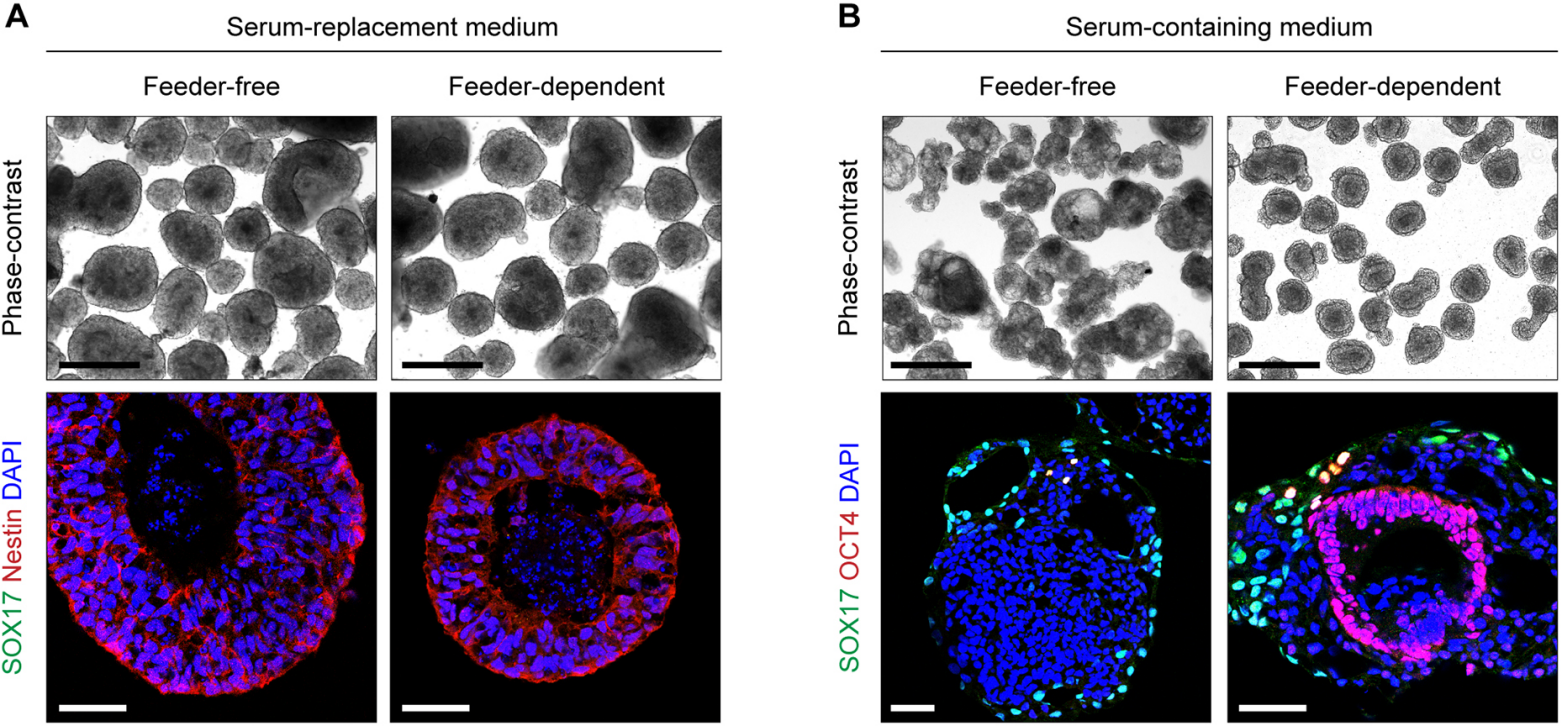
**Culture conditions impact the lineage outcome of hiPSC-derived embryoid bodies.** (A) Phase-contrast and immunohistochemistry images of hiPSC spheroids maintained in 20% serum-replacement medium for 5 days. Feeder-dependent spheroids were derived from hiPSCs cultured on a feeder layer of MEFs for at least one passage. (B) Day 5 spheroids in serum-containing medium. Images are representative of two independent cell culture experiments per condition. Scale bars: 500 µm (phase-contrast); 50 µm (fluorescence).

To establish an hiPSC-based model of basal lamina assembly, we sought to adapt a well-characterized 3D tissue culture method originally used with human embryonic stem cells (ESCs) (Ungrin et al., 2008). First, we used a Microwell plate to generate spheroids of hiPSCs and, following transfer of the spheroids into suspension culture, we tested multiple conditions for optimal ECM production. Spheroids grown in a standard knockout serum-replacement medium formed a cavitated core and differentiated into a Nestin^+^ neuroectodermal lineage (Fig. 1A). This result could be achieved with either feeder-free hiPSCs or with feeder-dependent hiPSCs, the latter of which were cultured on a feeder layer of mouse embryonic fibroblasts (MEFs) prior to spheroid formation.

We next tested the effect of serum on hiPSC spheroid differentiation, which has been used previously with mouse and human ESC spheroids (Ungrin et al., 2008; Li et al., 2017). In a serum-containing medium, spheroids from feeder-free hiPSCs consisted of an outer SOX17^+^ endodermal layer and a disorganized core of differentiated OCT4^−^ cells (Fig. 1B). These spheroids, as well as those discussed above that were maintained in serum-replacement medium, were mostly devoid of ECM as assessed by antibodies against the basal lamina protein laminin (data not shown).

**Fig. 2.**
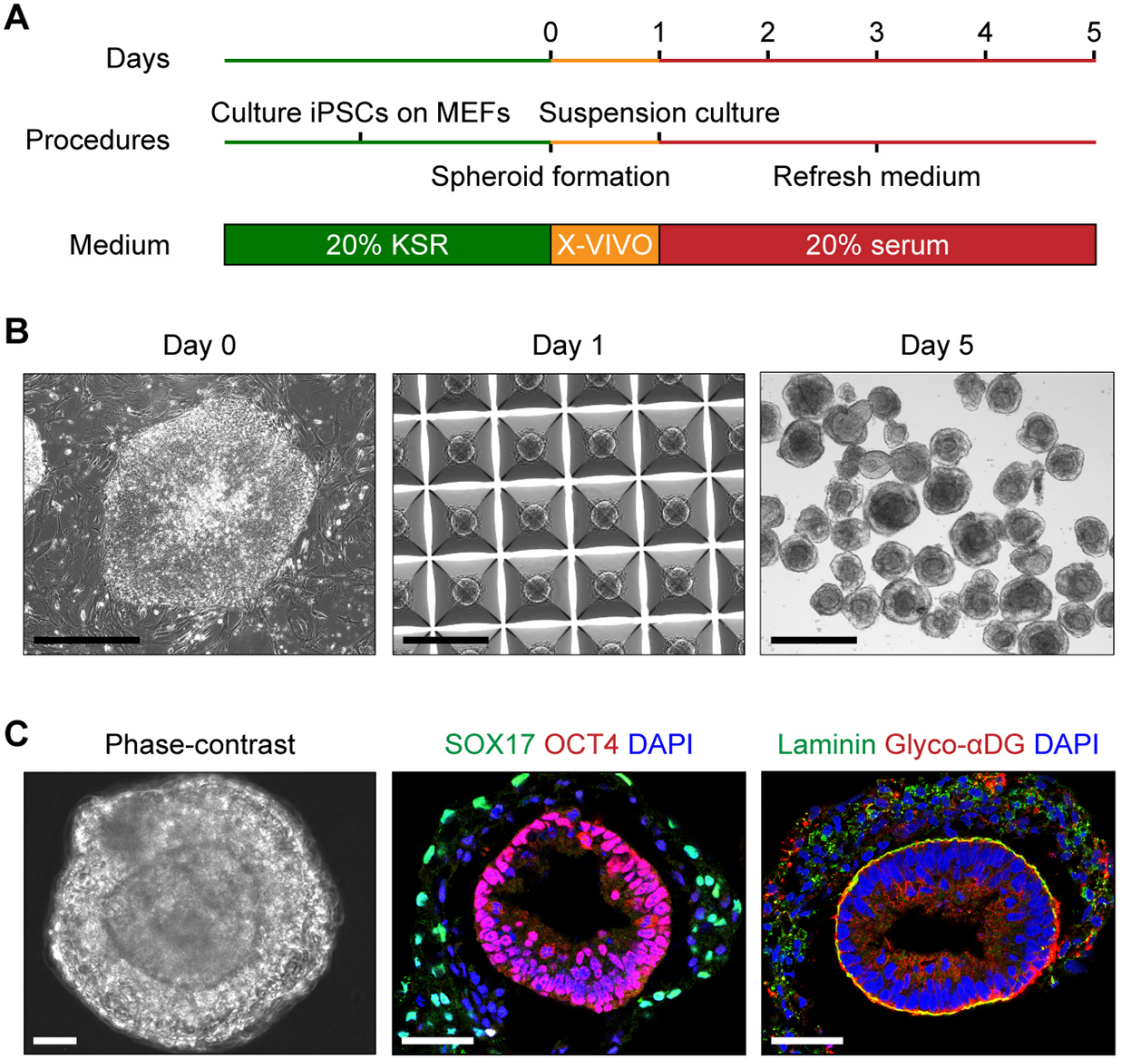
**Self-organization of ECM-containing embryoid bodies from hiPSCs.** (A) Schematic of embryoid body differentiation protocol from feeder-dependent hiPSCs. X-VIVO refers to X-VIVO 10 medium (see Materials and Methods). (B) Phase-contrast representation of embryoid body differentiation. hiPSCs were seeded on day 0 in a Microwell plate to form spheroids of roughly 2000 cells on day 1. The spheroids were then maintained in suspension culture until day 5. Scale bars: 500 µm. (C) Phase-contrast and immunohistochemistry images of day 5 embryoid bodies showing two distinct tissue domains, with a basal lamina in contact with the interior OCT4^+^ cells expressing glyco-αDG. Images are representative of three independent culture replicates. Scale bars: 50 µm.

In contrast, we found that feeder-dependent hiPSCs produced spheroids with two visibly partitioned domains when cultured in serum-containing medium (Fig. 1B and Fig. 2). These particular spheroids were characterized by a SOX17^+^ endodermal periphery and an OCT4^+^ epithelial core. The inner and outer tissue compartments were demarcated by a laminin-rich basal lamina (Fig. 2C). The SOX17^+^ endodermal cells were apparently responsible for ECM secretion, with disorganized aggregates of laminin visible only in the spheroid outer layer (Fig. 2C). The underlying core of OCT4^+^ cells appeared as a radially arranged epithelium and expressed the basal lamina receptor αDG, which was enriched at the basal lamina interface between the two tissue domains (Fig. 2C). Identical tissue patterning has been reported in spheroids derived from mouse and human ESCs (Li et al., 2002; Ungrin et al., 2008). The observed structure is thought to represent pre-gastrulation embryonic development, with an epiblast-like core and an outer layer of extra-embryonic endoderm. Because the hiPSC-derived spheroids produced with our protocol resemble this developmental stage, we refer to them hereafter as embryoid bodies.

### Derivation of hiPSCs from α-dystroglycanopathy patients

Because hiPSC-derived embryoid bodies express αDG and produce ECM apparently in the form of a basal lamina, we sought to apply this system for evaluating basal lamina phenotypes in α-dystroglycanopathy – a diverse spectrum of muscular dystrophies often occurring alongside brain malformation, characterized by ruptures in the basal lamina (Godfrey et al., 2011). We reprogrammed hiPSCs from dermal fibroblasts of three unrelated individuals with a genetic diagnosis of α-dystroglycanopathy. Each patient harbored predicted pathogenic mutations in a different gene required for the glycosylation of αDG: *LARGE*, *FKRP* or *POMT2* (Table S1). These three genes encode distinct glycosyltransferase enzymes, which catalyze the addition of specific sugars onto αDG matriglycan structures. POMT2 is partly responsible for initiating the installation of matriglycans by transferring an O-linked mannose to αDG (Manya et al., 2004). From this O-linked mannose, a series of other enzymes extend a complex polysaccharide, including a ribitol 5-phosphate added by FKRP (Gerin et al., 2016; Kanagawa et al., 2016; Praissman et al., 2016). Lastly, LARGE catalyzes the addition of a long repeating disaccharide, comprising xylose and glucuronic acid, which is the ECM ligand-binding element of matriglycan (Inamori et al., 2012; Briggs et al., 2016).

Clinical presentation for all three patients included delayed motor milestones, muscle weakness and cognitive impairments (Table S1). Overall, the patient with an *FKRP* mutation had the mildest clinical and muscle biopsy findings, while the patient with the *POMT2* mutations had the most severe findings. The patient with mutated *LARGE* has been reported in a previous publication (Meilleur et al., 2014). Brain magnetic resonance imaging showed minor white matter and structural abnormalities in the patients with mutated *LARGE* (Meilleur et al., 2014) and *POMT2* (data not shown). Hereafter, patients with these mutations are referred to simply as ‘FKRP’, ‘POMT2’ and ‘LARGE’ patients, respectively.

**Fig. 3.**
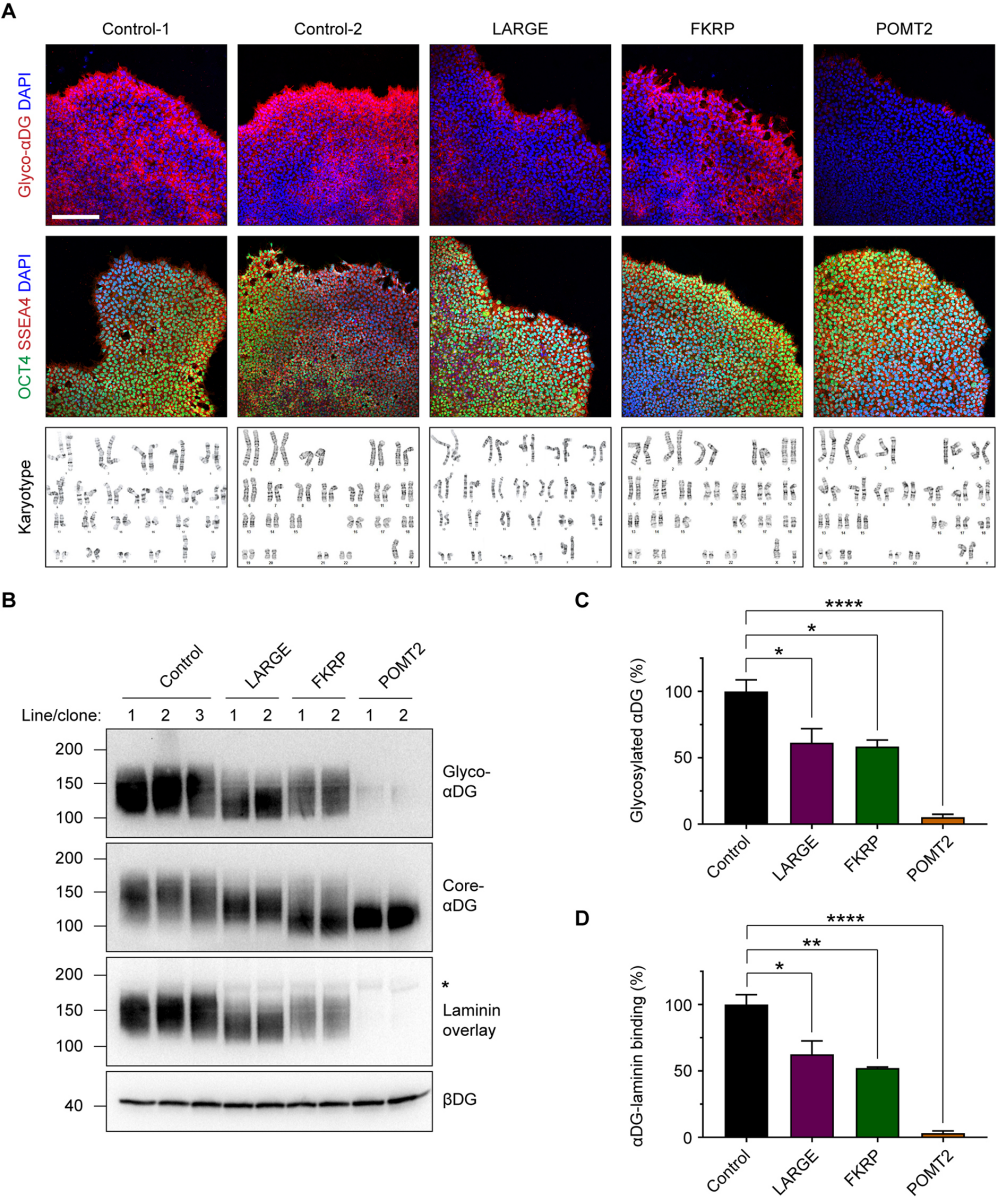
**α-Dystroglycanopathy patient hiPSCs express hypoglycosylated forms of αDG.** (A) Immunocytochemistry and karyotype analyses of control and α-dystroglycanopathy patient-derived hiPSCs. Scale bar: 200 µm. (B) Western blots on wheat germ agglutinin-enriched hiPSC protein lysates. βDG was used as a loading control. The asterisk indicates the molecular weight of endogenous laminin in the samples. Each lane represents one cell line (for controls) or one clone (for patients). (C) Quantification of western blots on glyco-αDG and the laminin overlay assay. Band intensity for each sample was normalized to βDG, and all samples are graphed as a percentage of the control. For glyco-αDG: control, 100.0±8.7%; LARGE, 61.4±10.5%; FKRP, 58.4±5.0%; POMT2, 5.3±2.1%. For laminin overlay: control, 100.0±7.5%; LARGE, 62.5±10.2%; FKRP, 52.3±0.7%; POMT2, 3.2±1.7%. Values expressed as mean±s.e.m. Three control cell lines and two clones per patient were used, *n*=6 independent cell culture replicates for control and *n*=3 per patient. Post hoc analysis using one-way ANOVA with Tukey's correction for multiple comparisons: *P<0.05, **P<0.01, ****P<0.0001.

Hypoglycosylation of αDG is the primary causative factor in the pathogenesis of α-dystroglycanopathy (Michele et al., 2002). To evaluate the glycosylation status of αDG in patient cells, we used the IIH6C4 antibody that specifically recognizes the glycosylated form of αDG (glyco-αDG). Based on a qualitative assessment, we found reduced immunofluorescence labeling with this antibody in our patient hiPSCs (Fig. 3A). Two hiPSC clones were evaluated for each patient. All cell lines had a normal karyotype, expressed pluripotent markers (Fig. 3A) and were capable of differentiating into the three germ layers (Fig. S1A–C).

To quantitatively confirm that patient cells express a hypoglycosylated form of αDG, we carried out western blots on hiPSC culture lysates using the IIH6C4 antibody (Ervasti and Campbell, 1991). Probing for glyco-αDG showed a reduction that roughly correlated with the clinical severity for each patient (Fig. 3B,C). Control hiPSCs expressed αDG glycoforms averaging ∼140 kDa, which is slightly less than is reported in human muscle (Jensen et al., 2015). Previous analysis suggests that low molecular weight forms of αDG – a consequence of fewer glycan structures – is a specific biochemical hallmark associated with disease severity (Goddeeris et al., 2013). Our study followed this pattern, with cells from the clinically mild FKRP patient expressing glyco-αDG of the same mass as controls but in reduced abundance. Glyco-αDG from the LARGE patient, who was of intermediate severity, showed a ∼30 kDa downward shift in molecular weight. hiPSCs from the severe POMT2 patient were virtually devoid of glyco-αDG.

To test the functional impact of these hypoglycosylated forms of αDG, we performed a laminin overlay assay to measure the affinity of αDG for one of its ECM ligands, laminin. All patient cell lines showed reduced αDG-laminin binding activity that closely matched their degree of αDG hypoglycosylation (Fig. 3B,D). Blotting with antibodies against the core peptide of αDG (core-αDG) and β-dystroglycan (βDG) indicated similar expression across all samples, demonstrating that the dystroglycan proteins are expressed but that αDG is hypoglycosylated in α-dystroglycanopathy patient hiPSCs (Fig. 3B).

A caveat of studying mutations in the *LARGE* gene is that there is a homologous glycosyltransferase, LARGE2, with the same enzymatic function (Inamori et al., 2013). Therefore, if *LARGE* and *LARGE2* are co-expressed in the same cell, LARGE2 could blunt the phenotype caused by LARGE loss-of-function. Based on this concern, we examined *LARGE* and *LARGE2* expression in control hiPSCs using a publicly available RNA expression database (https://stemcelldb.nih.gov/public.do) and our previously published hiPSC RNA sequencing data (GEO accession GSE139273) (Nickolls et al., 2020). We found that both datasets show similar expression levels of *LARGE* and *LARGE2* (12.66±1.0 TPM and 12.44±0.7 TPM, respectively; values expressed as mean±s.e.m. transcripts per million of *n*=4 samples from the GSE139273 database). This indicates that data obtained from α-dystroglycanopathy hiPSCs with *LARGE* mutations should be interpreted with caution, because LARGE2 enzymatic activity could be rescuing functional glycosylation of αDG. However, we suggest that the native expression level of *LARGE2* alone may be insufficient to compensate for LARGE in hiPSCs, given the significant hypoglycosylation of αDG in hiPSCs from the LARGE patient (Fig. 3B,C).

### Differentiation of embryoid bodies from α-dystroglycanopathy hiPSCs

Muscle, eye and brain abnormalities linked to basal lamina defects is a frequent finding in the α-dystroglycanopathies (Nakano et al., 1996; Ishii et al., 1997; Yamamoto et al., 1997, 2008; Saito et al., 1999; Goddeeris et al., 2013; Meilleur et al., 2014). Given that α-dystroglycanopathy patient hiPSCs exhibit the biochemical hallmark of the disease (i.e. hypoglycosylation of the basal lamina receptor αDG), we next asked whether patient embryoid bodies can synthesize basal lamina. To investigate potential disease-related phenotypes, we initially restricted our analysis to embryoid bodies from the LARGE patient and POMT2 patient, who had moderate and severe clinical findings, respectively.

**Fig. 4.**
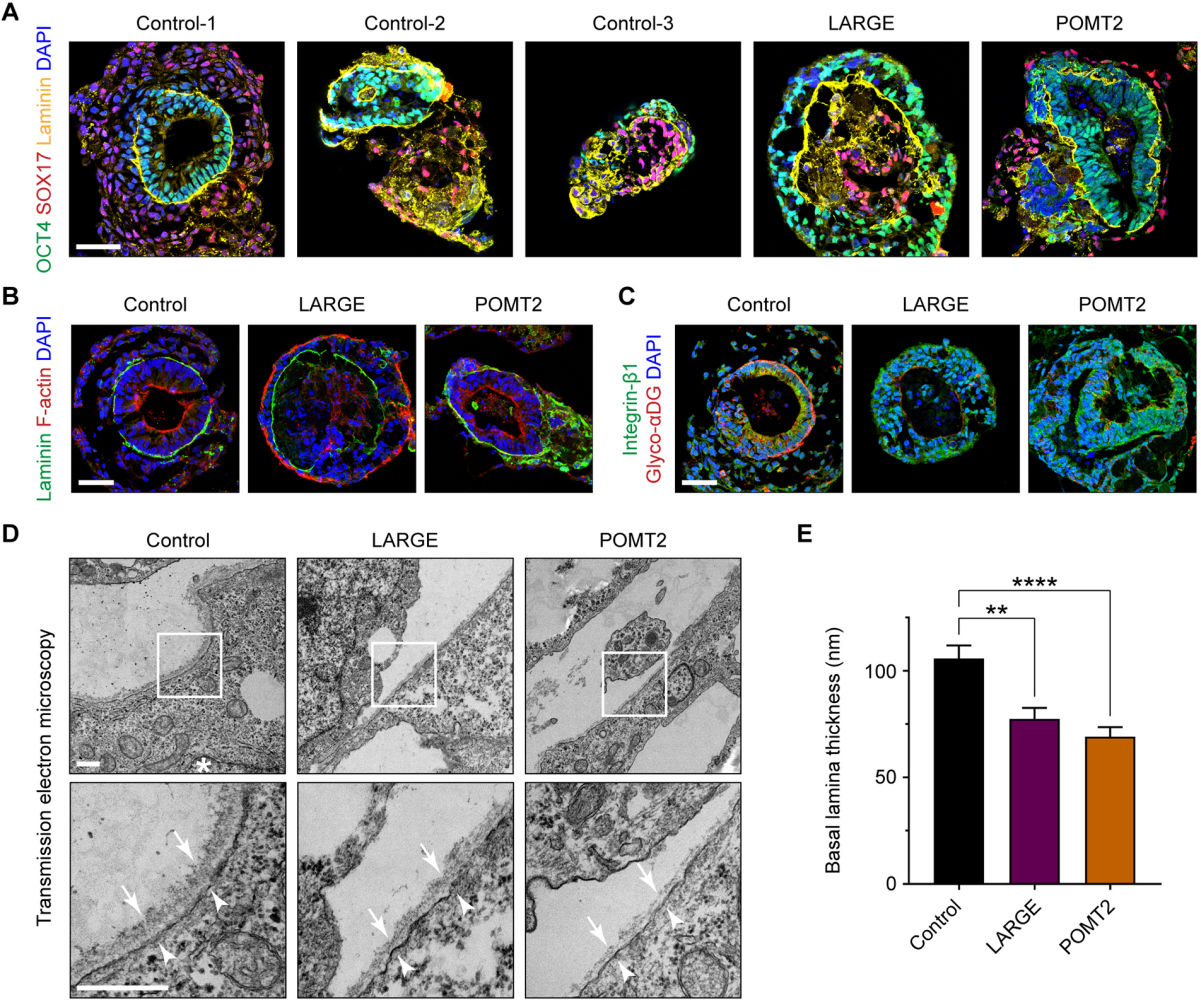
**Ultrastructural ECM defects in α-dystroglycanopathy patient embryoid bodies.** (A–C) Representative immunohistochemistry images of control and patient embryoid bodies at day 5 of differentiation. At least three independent differentiations were carried out on each of three control hiPSC lines and two hiPSC clones from the LARGE and POMT2 patients. Scale bars: 50 µm. (D) Transmission electron micrographs of embryoid body basal lamina. Asterisk, nucleus; arrows, basal lamina; arrowheads, plasma membrane. Scale bars: 500 nm. (E) Measurements of basal lamina thickness: control, 105.8±6.1 nm; LARGE, 77.5±5.1 nm; POMT2, 69.1±4.5 nm. Values shown as mean±s.e.m. Three control lines and two clones each from the LARGE and POMT2 patients were used. For each clone, micrographs from 2–3 independent differentiations were collected, and basal lamina was measured from *n*=35 (control), *n*=22 (LARGE) and *n*=23 (POMT2) separate regions throughout the cultures. Post hoc analysis with Kruskal–Wallis test and Dunn's correction for multiple comparisons: **P<0.01, ****P<0.0001.

We differentiated control and patient hiPSCs into embryoid bodies using the protocol described earlier. Embryoid bodies from all cell lines contained a basal lamina sandwiched by epithelial and endodermal compartments (Fig. 4A). There was morphological variation across cell lines, possibly related to genetic background or clonal differences. In particular, in embryoid bodies from the third control and from the LARGE patient, there was an occasional inversion of tissue layers such that the epithelial cells were on the exterior of the embryoid body.

Despite the heterogeneity between cultures, embryoid bodies from all control and patient hiPSC clones were similarly capable of assembling a laminin-rich basal lamina at the surface of the OCT4^+^ epithelium (Fig. 4A). Additionally, all embryoid bodies showed the expected morphology of epithelial polarity (Fig. 4B). OCT4^+^ cells were radially arranged and exhibited apicobasal polarity, with F-actin distributed on the cellular edge opposite from the basal lamina. Thus, at this resolution of analysis, we detected no major phenotypic difference between control and α-dystroglycanopathy embryoid bodies.

Consistent with our finding in undifferentiated hiPSCs, embryoid bodies from LARGE and POMT2 patients were minimally reactive to an antibody recognizing glyco-αDG. However, as shown previously in mouse embryoid bodies (Li et al., 2001, 2002), all embryoid bodies expressed integrin-β1, the primary β-subunit of integrin found in early development (Fig. 4C). Various integrin isoforms and αDG glycoforms can serve as laminin receptors, and the study of mouse embryoid bodies has shown functional redundancy between integrins and αDG in anchoring ECM molecules to the cell membrane (Li et al., 2017). This likely explains the ability of embryoid bodies from α-dystroglycanopathy patients to assemble a basal lamina in the absence of αDG receptor function.

### Ultrastructural basal lamina defects in α-dystroglycanopathy embryoid bodies

Previous studies of tissue from α-dystroglycanopathy patients and mice have revealed ultrastructural ECM defects (Ishii et al., 1997; Goddeeris et al., 2013). To visualize embryoid body ECM ultrastructure, we employed transmission electron microscopy on thin sections of control and patient samples. In electron micrographs, basal lamina from control embryoid bodies was visible as a fibrous layer at the epithelial cell surface, roughly 100 nm thick (Fig. 4D). The overlying endodermal cell, some distance away, was always devoid of basal lamina. The basal lamina was composed of a ‘lamina lucida’ and a ‘lamina densa’ compartment. The lamina lucida – a thin electron-light layer at the epithelial plasma membrane – is believed to be spanned by the laminin long arm bound to its cell surface receptors, αDG and integrin (Schittny et al., 1988). The lamina densa – a thicker fibrous layer of electron-dense material just above the lamina lucida – comprises laminin cross-linking arms, perlecan, nidogen and COLIV. However, the existence or extent of the lamina lucida may also be an artifact of our sample dehydration method (Chan and Inoue, 1994).

The epithelial cells of embryoid bodies showed typical features of lateral, apical and basal polarization. We observed electron-dense tight junctions at cell–cell borders, and microvilli decorated the epithelial cell apical aspect facing the embryoid body lumen (Fig. S2). Nuclei were polarized toward the basal aspect of epithelial cells in contact with the basal lamina (Fig. 4D). Occasionally, filamentous matrix could be seen in the extracellular space between endodermal and epithelial cells, but it was rarely attached to the basal lamina itself (Fig. S2). Therefore, the ECM structures in our embryoid bodies meet the criteria for an epithelial basal lamina. However, they cannot be categorized as a complete basement membrane, which requires an adjoined layer of ‘lamina reticularis’ fibrillar collagens (Sanes, 1982).

In LARGE and POMT2 embryoid bodies, the basal lamina was noticeably thinner owing to a reduction of material in the lamina densa (Fig. 4D). Specifically, in POMT2 embryoid bodies, the basal lamina occasionally contained nanoscopic discontinuities. The basal lamina in control embryoid bodies measured 105.8±6.1 nm thick. LARGE and POMT2 basal lamina were significantly thinner at 77.5±5.1 and 69.1±4.5 nm, respectively (mean±s.e.m., *P*=0.0022 and *P*<0.0001) (Fig. 4E).

**Fig. 5.**
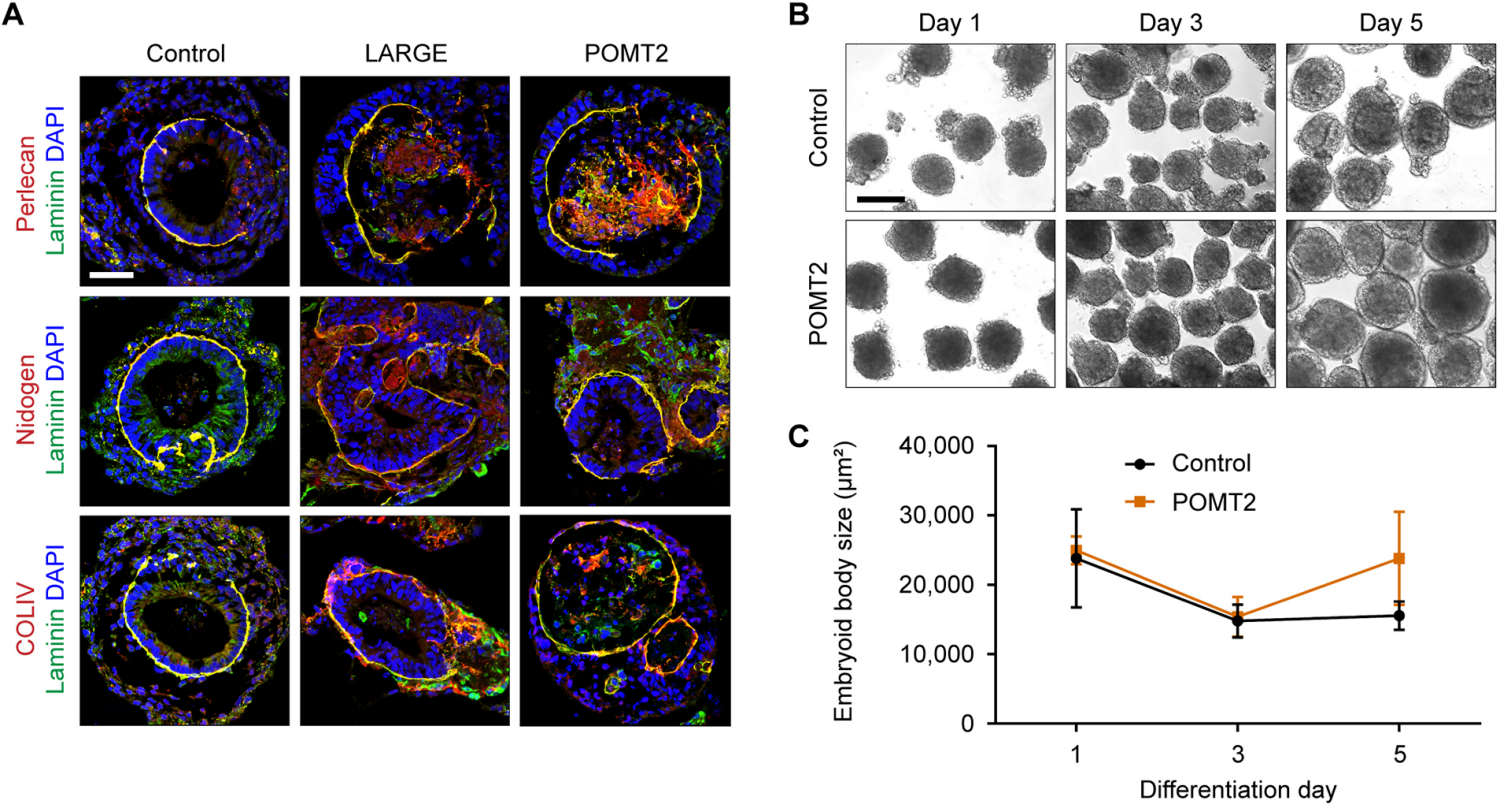
**ECM localization and growth characteristics of α-dystroglycanopathy embryoid bodies.** (A) Representative antibody labeling to assess co-localization of laminin with other basal lamina constituents from two independent cell culture experiments per cell line. Embryoid bodies from three controls and two clones per patient were used. Scale bar: 50 µm. (B) Phase-contrast images of embryoid body differentiation on days 1, 3 and 5. Scale bar: 100 µm. (C) Quantification of embryoid body size over time: control, 23,813±7078 μm^2^ (day 1), 14,797±2376 μm^2^ (day 3), 15,544±2030 μm^2^ (day 5); POMT2, 24,981±2010 μm^2^ (day 1), 15,384±2869 μm^2^ (day 3), 23,821±6718 μm^2^ (day 5). Measurements expressed as mean±s.d. from *n*=3 differentiations based on averaged cross-sectional area in phase-contrast images. At least 75 embryoid bodies per line were analyzed during each differentiation. Using one-way ANOVA with Tukey's correction for multiple comparisons, no statistical significance was found between control and patient at any time point (*P*>0.05).

We considered whether a mislocalization of certain ECM molecules might explain the reduced thickness of patient basal lamina. In addition to laminin, αDG directly binds to perlecan, which, in turn, crosslinks nidogen and collagen type IV (COLIV) to form the basal lamina (Peng et al., 1998; Talts et al., 1999). Using immunohistochemistry, we examined the localization of these basal lamina components in embryoid bodies (Fig. 5A). Morphologically mature embryoid bodies from control, LARGE and POMT2 hiPSCs similarly had COLIV, nidogen and perlecan associated with laminin, suggesting a normal composition of ECM molecules in the basal lamina. Overall, there were no consistently detectable differences in the staining pattern between control and α-dystroglycanopathy embryoid bodies.

Because our embryoid body differentiation protocol requires co-culture of hiPSCs with MEFs, we reasoned that abundant fibroblast ECM secretion could be influencing or masking basal lamina phenotypes. To evaluate this possibility, we labeled control and POMT2 hiPSCs and embryoid bodies with antibodies against human nuclear antigen (HuNu). In feeder-dependent hiPSC cultures, hiPSCs were clearly distinguished by HuNu expression, whereas MEFs were heavily labeled by laminin antibodies (Fig. S3A). In embryoid bodies, hiPSCs and MEFs self-segregated within 24 h of spheroid formation, with MEFs budding off and ultimately detaching from the differentiating embryoid body 2–4 days before basal lamina formation (Fig. S3B). Because all analyses were conducted on day 5 embryoid bodies, we believe the presence of MEFs is not a significant confounding factor in the above experiments.

Over the course of differentiation, we also observed that embryoid bodies were remarkably static in size, and there was no significant difference between control and POMT2 embryoid body surface area (Fig. 5B,C). This contrasts greatly with brain and muscle tissue, which undergo significant size expansion and mechanical strain during embryonic development and muscle contraction, respectively (Hu et al., 2007; Han et al., 2009). Thus, the embryoid body differentiation protocol serves as a simplified model for patient-specific basal lamina assembly, without the additional variables of tissue movement and growth. In this system, α-dystroglycanopathy embryoid bodies with mutations in *LARGE* or *POMT2* can synthesize a basal lamina of apparently typical molecular composition but with abnormal thinness. We suggest that this ultrastructural phenotype is only observable with electron microscopy because either: (1) traditional immunofluorescence microscopy lacks the spatial resolution to discriminate a ∼30 nm change in thickness; or (2) most embryoid bodies contain additional secreted ECM between the endodermal and epithelial layers that, while not part of the basal lamina itself, may be indistinguishable by immunofluorescence (Fig. S2). Overall, our data corroborate published results demonstrating that αDG is generally dispensable for initial basal lamina formation but has a role in its structural maturation or maintenance (Cote et al., 1999; Li et al., 2002; Moore et al., 2002).

### Impaired laminin accumulation on endoderm-free α-dystroglycanopathy embryoid bodies

**Fig. 6.**
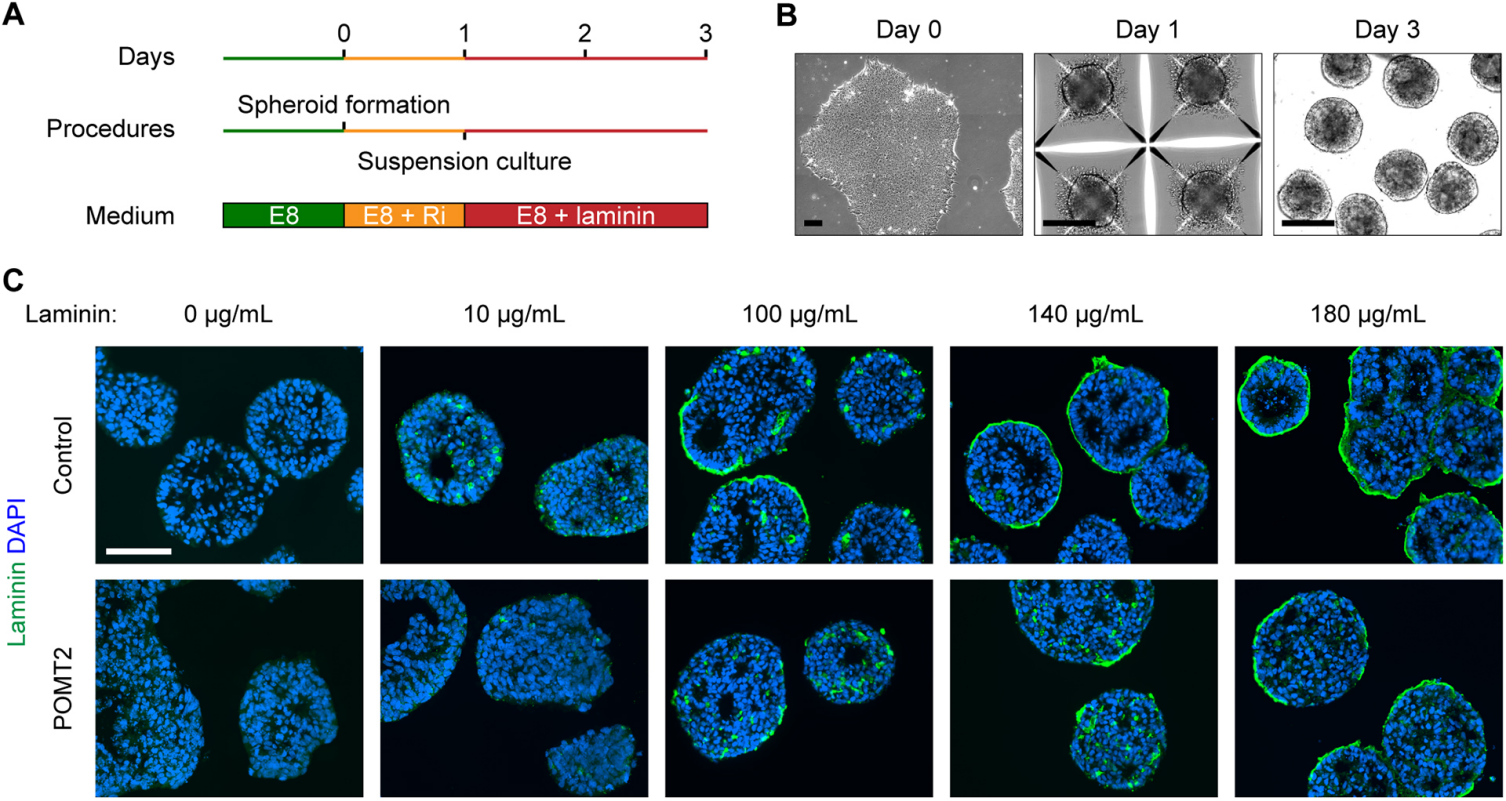
**Accumulation of exogenous laminin on endoderm-free embryoid bodies.** (A,B) Schematic and phase-contrast images of endoderm-free embryoid body culture protocol. Feeder-free hiPSCs were seeded on day 0 in Microwell plates to form spheroids of roughly 1000 cells by day 1. The spheroids were transferred to suspension culture supplemented with laminin for 48 h. (C) Immunohistochemistry demonstrating the effect of increasing laminin concentration on endoderm-free embryoid bodies. Embryoid bodies were supplemented with varying concentrations of laminin on day 1 and collected for analysis 48 h later. Two independent cell culture replicates were performed per cell line. Scale bars: 100 µm.

Knockout of dystroglycan in mouse embryonic stem cells and neural stem cells has been reported to reduce laminin association at the cell surface, which is a prerequisite for establishment of the basal lamina (Henry and Campbell, 1998; Zhang et al., 2013). Because we observed thinner basal lamina in α-dystroglycanopathy patient embryoid bodies, we next investigated whether this phenotype might be linked to a reduced ability to recruit laminin. We developed an embryoid body culture protocol that both prevents the formation of laminin-secreting endoderm and obviates the need for MEF co-culture (Fig. 6A,B). This eliminated the major sources of ECM and allowed for precise control of laminin concentration by exogenous supplementation in the growth medium.

In the absence of exogenously added laminin, endoderm-free embryoid bodies were virtually devoid of laminin (Fig. 6C). We supplemented various concentrations of laminin to control embryoid bodies and inspected the cultures after 48 h. Amounts greater than 100 µg/ml resulted in a thin layer of laminin at the surface of some embryoid bodies (Fig. 6C). POMT2 embryoid bodies – from the most severe α-dystroglycanopathy patient – showed a striking absence of accumulated surface laminin except in the highest concentration tested, 180 µg/ml. To avoid ceiling and floor effects, we used 140 µg/ml laminin in subsequent experiments, because approximately half of the control embryoid body surface area was bound by laminin at this concentration (Fig. 6C).

**Fig. 7.**
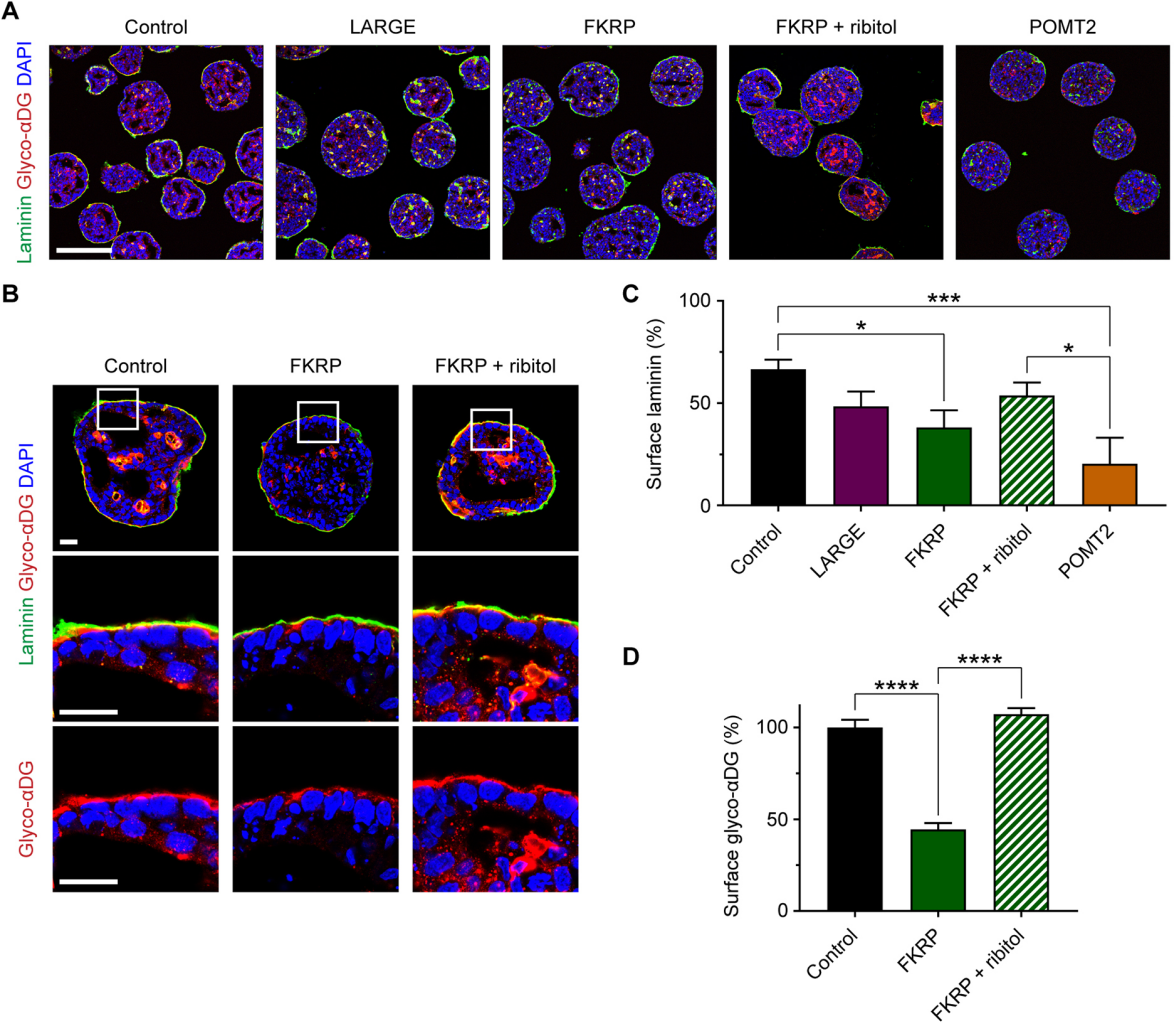
**Patient-specific differences in laminin accumulation and response to ribitol treatment.** (A) Immunohistochemistry on day 3 endoderm-free embryoid bodies supplemented for 48 h with exogenous laminin. Scale bar: 200 µm. (B) High-magnification images to assess the effect of ribitol treatment on the localization and glycosylation of αDG in FKRP patient embryoid bodies. Scale bar: 25 µm. (C) Quantification of the percentage embryoid body surface area covered by laminin: control, 66.6±4.7%; LARGE, 48.4±7.3%; FKRP, 38.2±8.3%; FKRP+ribitol, 53.8±6.3%; POMT2, 20.5±5.2%. Values expressed as mean±s.e.m. Three controls and two clones per patient were analyzed across *n*=12 (control), *n*=6 (LARGE and POMT2), and *n*=9 (FKRP and FKRP+ribitol) independent cell culture differentiations. Post hoc analysis with one-way ANOVA and Tukey's correction for multiple comparisons: *P<0.05, ***P<0.001. (D) Quantification of glyco-αDG staining intensity at the embryoid body surface: control, 100.0±4.2%; FKRP, 44.5±3.5%; FKRP+ribitol, 107.2±3.4%. Values normalized to a percentage of the controls and expressed as mean±s.e.m. Two controls and two FKRP clones were analyzed across three cell culture differentiations to measure *n*=54 surface regions of 50 µm length per condition. Post hoc analysis with one-way ANOVA and Tukey's correction for multiple comparisons: ****P<0.0001.

We tested endoderm-free embryoid bodies from control subjects and LARGE, FKRP and POMT2 α-dystroglycanopathy patients for their capacity to accumulate laminin (Fig. 7A,B). Compared with controls, which recruited laminin on 66.6±4.7% of surfaces, POMT2 embryoid bodies showed 20.5±5.2% surface laminin (mean±s.e.m., *P*=0.0006) (Fig. 7C). In contrast, LARGE and FKRP embryoid bodies recruited 48.4±7.3% and 38.2±8.3% laminin, respectively, relative to controls (*P*=0.319 and *P*=0.0127) (Fig. 7C). Altogether, these data show a deficit in the surface accumulation of laminin on α-dystroglycanopathy patient embryoid bodies.

### Ribitol treatment promotes functional glycosylation of αDG in FKRP patient embryoid bodies

FKRP is a glycosyltransferase enzyme that catalyzes the addition of ribitol phosphate to αDG (Gerin et al., 2016; Kanagawa et al., 2016; Praissman et al., 2016). This is an essential enzymatic step for the post-translational installation of matriglycans on αDG, which are the structural basis for ligand binding to αDG (Briggs et al., 2016). Recently, dietary supplementation with ribitol was shown to improve muscle phenotypes in a mouse model of FKRP-related α-dystroglycanopathy (Cataldi et al., 2018).

To determine the effect of ribitol on FKRP hiPSCs, we supplemented the culture medium with 3 mM ribitol daily and harvested the cells 72 h later. This dosage was based on previous reports of efficacious treatment with 3 mM ribitol or CDP-ribitol in ISPD mutant α-dystroglycanopathy cell cultures (Gerin et al., 2016; Kanagawa et al., 2016). Ribitol treatment of FKRP hiPSCs greatly increased the abundance of glyco-αDG and resulted in a ∼20 kDa upward shift in molecular weight (Fig. S4A). This change was accompanied by a comparable increase in laminin binding to αDG. In addition to ribitol phosphate, FKRP is known to transfer glycerol phosphate onto αDG, which inhibits its functional glycosylation. However, we found that treating control and FKRP hiPSCs with 3 mM glycerol daily for 72 h had no discernible effect on αDG glycosylation (Fig. S4B).

Another candidate experimental therapy – overexpression of the LARGE enzyme – has been shown to restore αDG function in a variety of α-dystroglycanopathies caused by mutations in *FKRP*, *FKTN*, *POMGNT1* and *POMT1* (Barresi et al., 2004; Yu et al., 2013; Vannoy et al., 2014). To similarly examine the specificity of ribitol treatment, we tested its effect on the remaining α-dystroglycanopathy patient hiPSCs. We found that ribitol-treated LARGE and POMT2 hiPSCs were unchanged in the quantity and molecular weight of glyco-αDG (Fig. S4A).

Because ribitol treatment resulted in a specific and profound improvement to glyco-αDG in FKRP hiPSCs, it was next assessed on FKRP endoderm-free embryoid bodies. hiPSCs were treated with 3 mM ribitol daily starting 72 h before embryoid body formation and continued throughout the experiment. Treatment of FKRP embryoid bodies caused a marked increase in the staining intensity of glyco-αDG (Fig. 7A,B). However, with microscopy-based methods of measurement, we did not find a statistically significant change in surface laminin accumulation between ribitol-treated FKRP embryoid bodies (53.8±6.3%) and untreated FKRP embryoid bodies (38.2±8.3%; mean±s.e.m., *P*=0.414) (Fig. 7C). Importantly though, at laminin contact points, glyco-αDG staining intensity was upregulated to the same level as controls (ribitol-treated FKRP: 107.2±3.4%; untreated FKRP: 44.5±3.5%; normalized to control glyco-αDG, mean±s.e.m., *P*<0.0001) (Fig. 7B,D).

**Fig. 8.**
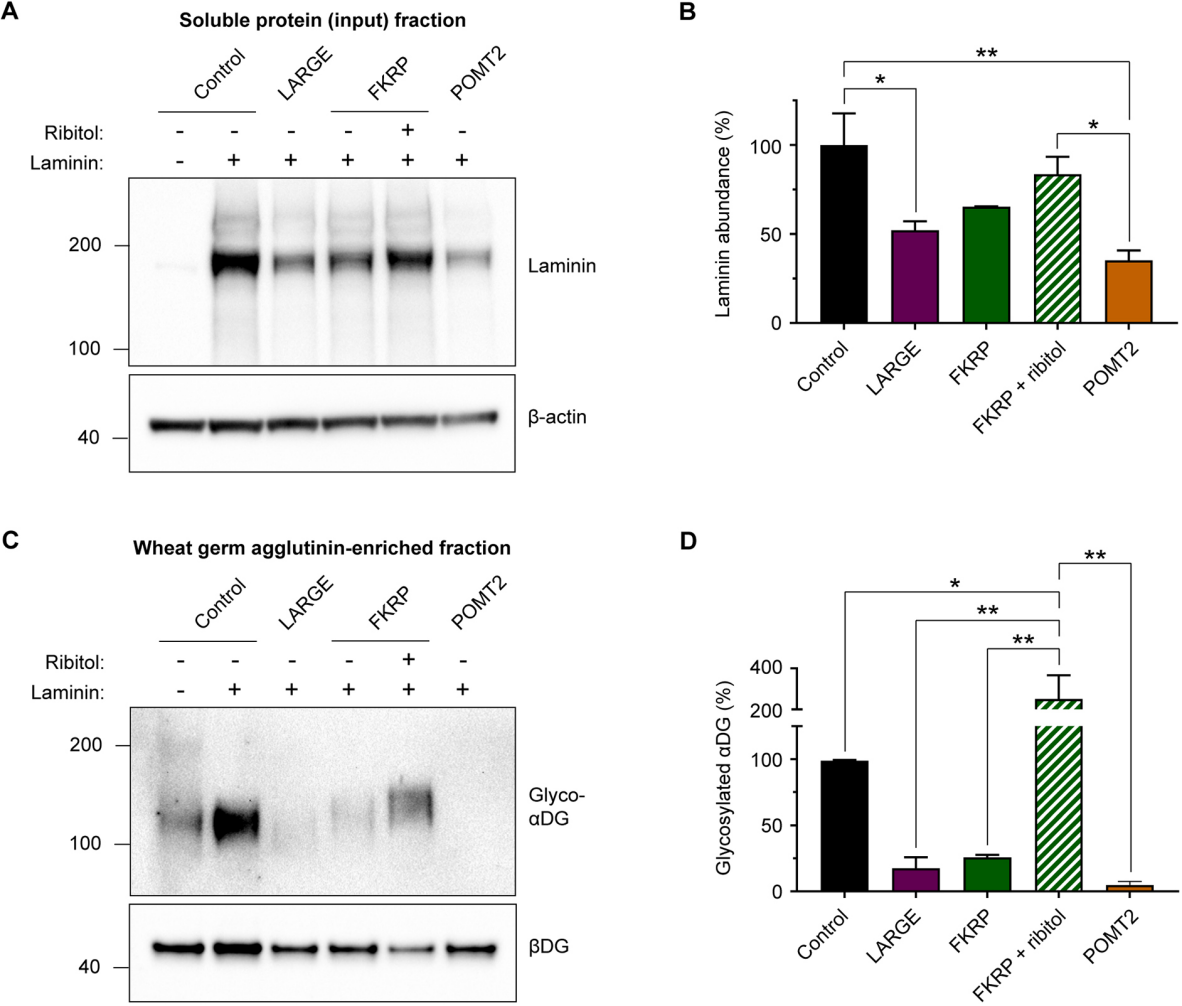
**Western blot analysis of endoderm-free embryoid bodies.** (A) Representative western blot measurement of laminin abundance in the input soluble protein fraction (not wheat germ agglutinin-enriched) from culture lysates of day 3 endoderm-free embryoid bodies. (B) Quantification of laminin band intensity: control, 100.0±17.8%; LARGE, 52.2±4.9%; FKRP, 65.4±0.2%; FKRP+ribitol, 83.6±5.7%; POMT2, 35.2±3.2%. Values expressed as mean±s.e.m. of *n*=3 independent cell culture replicates. Each sample was normalized to β-actin, and all samples are plotted as a percentage of control. Post hoc analysis with one-way ANOVA and Tukey's correction for multiple comparisons: *P<0.05, **P <0.01. (C) Representative western blot of glyco-αDG in wheat germ agglutinin-enriched culture lysates from day 3 endoderm-free embryoid bodies. (D) Quantification of glyco-αDG band intensity: control, 100.0±0.6%; LARGE, 17.6±8.3%; FKRP, 25.9%±1.9%; FKRP+ribitol, 250.8±67.3%; POMT2, 4.9±1.5%. Values plotted as mean±s.e.m. of *n*=3 independent cell culture replicates. Each sample was normalized to βDG, and all samples are expressed as a percentage of control. Post hoc analysis with one-way ANOVA and Tukey's correction for multiple comparisons: *P<0.05, **P<0.01.

We next performed quantitative western blot analyses of the endoderm-free embryoid body cultures. The abundance of laminin in the soluble protein fraction was reduced in α-dystroglycanopathy patient samples relative to controls, with the severe POMT2 patient being the lowest (LARGE, 52.2±4.9%; FKRP, 65.4±0.2%; POMT2, 35.2±3.2%; values mean±s.e.m. percentage of control) (Fig. 8A,B). Ribitol treatment of the FKRP patient embryoid bodies apparently increased the accumulation of laminin (83.55±5.7%; mean±s.e.m. percentage of control), but it did not reach statistical significance when compared with untreated FKRP.

We then examined the amount of glyco-αDG in endoderm-free embryoid bodies via western blotting. Interestingly, compared with negative control cultures lacking laminin, addition of exogenous laminin seemed to increase glyco-αDG content in healthy control embryoid bodies (Fig. 8C; data not shown). This phenomenon was not quantitatively evaluated or investigated further in this study, but it suggests that the presence of laminin might stabilize glyco-αDG turnover or promote its expression. In LARGE, FKRP and POMT2 patient embryoid bodies, glyco-αDG was substantially reduced (Fig. 8C,D) in a pattern that largely corresponded with that of the earlier western blots from iPSCs (Fig. S4). Further, ribitol treatment significantly upregulated glyco-αDG in the FKRP patient embryoid bodies (Fig. 8C,D). Together, these results indicate ribitol treatment as a viable therapeutic strategy for functionally upregulating αDG glycosylation in FKRP patient tissue.

## DISCUSSION

Our results provide an approach for evaluating patient-specific basal lamina *in vitro*. We applied this method to study patients with α-dystroglycanopathy. We found that patient hiPSC-derived embryoid bodies recapitulate the clinical disease spectrum and exhibit the ECM defects seen in animal models. Finally, this system allowed us to evaluate the efficacy of ribitol supplementation – a recent candidate therapeutic for the α-dystroglycanopathies – in patient samples of different genotypes.

Embryoid body-based methods, which essentially involve culturing pluripotent stem cells in 3D aggregates, are widely used in stem cell research. Typical applications include evaluating the pluripotency of new cell lines or as an intermediate stage during differentiation toward specific lineages (Kurosawa, 2007). Under strict growth conditions, embryoid bodies from mouse and human embryonic stem cells (ESCs) can exquisitely self-organize into structures mimicking the pre-gastrulation embryo. These have served as models for developmental events – epiblast polarization and ECM formation – that are required for tissue genesis (Li et al., 2003).

Our hiPSC-derived embryoid bodies resemble their ESC-derived counterparts by similarly requiring serum-containing media and MEF co-culture to form polarized epiblast, basal lamina and extra-embryonic endoderm. It remains unclear why these conditions uniquely enable such self-organization. Bone morphogenetic proteins (BMPs), which are abundant in serum (Herrera and Inman, 2009), are known to induce extra-embryonic endoderm differentiation (Graham et al., 2014). Co-culture with MEFs can also influence the lineage bias of ESCs (Lippmann et al., 2014). Thus, it may be a combination of soluble factors from serum and MEFs that promotes the simultaneous existence of endoderm and epiblast cell populations in our hiPSC-derived embryoid bodies.

Dual genetic deletion of αDG and integrin-β1 prevents basement membrane formation and epithelial polarization in mouse ESC-derived embryoid bodies. Because these two receptors have overlapping roles, expression of one can partly rescue the function of the other (Li et al., 2017). By studying α-dystroglycanopathy patient embryoid bodies, we extend these findings to show that a moderate reduction in αDG receptor activity – with normal expression of both αDG and integrin-β1 core proteins – is linked to a subtly thinner basal lamina that may underlie disease pathogenesis.

A thin and discontinuous basal lamina has been reported in the muscle of α-dystroglycanopathy patients (Ishii et al., 1997; Yamamoto et al., 1997), similar to our observation in patient embryoid bodies. Also, the retina inner limiting basement membrane is thin, patchy and less stiff in *Pomgnt1*-null mice (Hu et al., 2010; Zhang et al., 2013). One caveat of our hiPSC-derived embryoid body system is that it seems to recapitulate only the basal lamina. It is unclear how the observed basal lamina defects might translate to the formation of a full basement membrane. The specific structural and molecular deficits underlying such abnormally thin basal lamina are still unknown. Furthermore, the microscopy methods employed in our study and others are not able to distinguish between reduced basal lamina assembly (i.e. ordered matrix polymerization) and reduced ECM accumulation (i.e. disordered matrix aggregation) and would require additional techniques to confirm (e.g. high angle platinum/carbon shadowing).

In contrast to the above findings, basement membranes in dystroglycan-null mouse embryoid bodies, and in the muscle of *Large* mutant mice and certain human patients, are thicker than controls, sometimes with mislocalized ECM components (Li et al., 2002; Goddeeris et al., 2013). These dichotomous observations might be related to differences in ECM recruitment, organization and maintenance at the cell surface, as a consequence of reduced αDG receptor activity but under specific tissue and disease state circumstances. One study reported a mixture of thinned and duplicated basal lamina in different regions of α-dystroglycanopathy patient muscle (Vajsar et al., 2000). Therefore, whether the diseased basal lamina is thin and patchy, or thick and duplicated, may vary due to local tissue conditions.

The pathogenic mechanism of brain malformation in the α-dystroglycanopathies is still not fully understood. During brain development, the human cerebral cortex undergoes massive expansion and folding in the third trimester (Garcia et al., 2018). This rapid increase of surface area likely places mechanical strain on the brain's basement membrane casing, necessitating timely ECM remodeling to accommodate the larger area. In mice, genetic deletion of ECM genes or hypoglycosylation of αDG both result in basement membrane rupture and ensuing tissue malformation during this most rapid phase of brain development (Halfter et al., 2002; Nakagawa et al., 2015; Booler et al., 2016; Sudo et al., 2018). These data present αDG as one of several laminin receptors that contributes to the efficient organization of ECM molecules into a coherent basement membrane.

Our result – that endoderm-free patient embryoid bodies show diminished laminin accumulation – corroborates evidence that αDG-deficient cells have reduced ECM-binding kinetics that might render basement membranes susceptible to mechanically induced deformation (Henry and Campbell, 1998; Zhang et al., 2013). Crucially, we found that embryoid bodies undergo minimal volumetric growth. This could explain the relatively mild phenotypes in embryoid bodies of even patients with the most severe α-dystroglycanopathy symptoms. The human brain specifically undergoes greatest expansion in the occipital, temporal and lateral parietal cortices (Garcia et al., 2018). Such regional growth dynamics suggest one possible basis for the spatial arrangement of the typical cortical malformation in the α-dystroglycanopathies, which are fundamentally due to breaches in basement membrane integrity, resulting in cellular over-migration beyond the confines of the basement membrane (Aida et al., 1996; Clement et al., 2008; Meilleur et al., 2014; Yoshioka et al., 2017). These particular growth dynamics may also underlie some of the differences between human patients and mouse models (Booler et al., 2015).

There is currently no effective treatment for the α-dystroglycanopathies, and the diversity of underlying genetic causes for the disease presents a challenge for targeted therapies. Supplementation of the sugar alcohol ribitol has recently emerged as a promising therapeutic for specific classes of α-dystroglycanopathy. In mammalian cells, the enzyme ISPD synthesizes CDP-ribitol from ribitol, which is then attached to αDG by the glycosyltransferases FKTN and FKRP (Gerin et al., 2016; Kanagawa et al., 2016; Praissman et al., 2016). This enzymatic process is a critical step in constructing the laminin-binding glycan of αDG.

Treatment of ISPD mutant cells with ribitol or CDP-ribitol promotes αDG glycosylation (Gerin et al., 2016; Kanagawa et al., 2016), and dietary ribitol supplementation rescues muscle phenotypes in *Fkrp* mutant mice (Cataldi et al., 2018). Here, we extend this concept from animal models to a human patient-derived system. We found specific efficacy of ribitol on FKRP patient hiPSCs, as evidenced by a complete rescue of glycosylated laminin-binding αDG. There was no detectable effect on LARGE and POMT2 patient cells, as would be expected from the location of these genetic forms in the pathway of αDG glycosylation. In FKRP endoderm-free embryoid bodies, ribitol treatment significantly upregulated glyco-αDG and slightly increased the accumulation of laminin at the embryoid body surface. However, the measurement of laminin accumulation cannot be confidently interpreted, as it did not reach statistical significance over untreated FKRP embryoid bodies. We speculate that either: (1) ribitol treatment has little effect on cell surface laminin accumulation in this *in vitro* system; or (2) the assay might lack the complexity or sensitivity to further detect functional improvements in an already relatively mild FKRP patient.

The FKRP patient in this study harbors a L276I mutation, the most common variant in the FKRP-related α-dystroglycanopathies (Frosk et al., 2005). We speculate that ribitol supplementation may be a rational and effective treatment, in particular for mild-to-moderate FKRP and FKTN patients with residual ribitol transferase function that can be boosted by the additional supply of substrate. It remains to be investigated whether ribitol would also benefit additional groups of α-dystroglycanopathy patients. Collectively, these data establish a system to interrogate basal lamina structure and ECM receptor function in patient tissue, expanding the options for personalized phenotyping and drug evaluation in the α-dystroglycanopathies.

## MATERIALS AND METHODS

### Generation of hiPSC lines

Written informed consent for patient participation was obtained by a qualified investigator (protocol 12-*N*-0095 approved by the National Institute of Neurological Disorders and Stroke, National Institutes of Health). α-Dystroglycanopathy patient hiPSCs were reprogrammed from dermal fibroblasts using an hOKSML mRNA reprogramming kit (Stemgent, 00-0067). Control-1 hiPSCs were reprogrammed in the same manner from BJ foreskin fibroblasts (ATCC, CRL-2522). Immunocytochemical validation of germ layer differentiation was performed off-site (Stemgent). Control-2 hiPSCs were reprogrammed from control foreskin fibroblasts (ATCC, CRL-2097) using the CytoTune-iPS 2.0 Sendai reprogramming kit (Thermo Fisher, A16517). Control-3 hiPSCs (NC15) were previously generated by lentiviral reprogramming of adult dermal fibroblasts (Grunseich et al., 2014). Karyotype analysis was performed after at least 10 passages (WiCell), and all cell lines were routinely tested for mycoplasma contamination (LT07-118, Lonza).

### hiPSC culture and supplementation

hiPSCs were maintained with daily changes of E8 medium (Thermo Fisher, A1517001) on tissue culture-treated polystyrene plates coated with Matrigel (Corning, 354277) and passaged every 4–6 days using ReLeSR (STEMCELL Technologies, 05872). For the sugar alcohol supplementation experiments, ribitol (Sigma, A5502) or glycerol (Sigma, G2025) was added to the growth medium at a final concentration of 3 mM. Supplementation was performed with daily medium changes in hiPSCs and every-other-day medium changes in embryoid bodies. For embryoid body experiments, the hiPSCs were pre-treated with ribitol for one passage before embryoid body formation, and ribitol treatment was continued throughout the embryoid body culture.

### Embryoid body differentiation

Differentiation of hiPSC-derived embryoid bodies was performed essentially as described previously for human embryonic stem cells (Ungrin et al., 2008). At least one passage before differentiation, hiPSCs were transitioned to MEF co-culture. The MEFs (Millipore, PMEF-CF) were seeded at 30,000 cells/cm^2^ on plates coated with gelatin (STEMCELL Technologies, 07903) and maintained in serum-containing medium consisting of KO-DMEM (10829-018), 20% FBS (26140-079), 100 µM non-essential amino acids (11140-050), 2 mM GlutaMAX (35050-061) and 55 µM β-mercaptoethanol (21985-023) (all from Invitrogen).

hiPSCs were dissociated with ReLeSR and plated on MEFs in knockout serum replacement medium consisting of KO-DMEM, 20% KSR (Invitrogen, 10828-028), 100 µM non-essential amino acids, 2 mM GlutaMAX, 55 µM β-mercaptoethanol, 10 ng/ml bFGF (Thermo Fisher, 233-FB-025), and 10 µM Y-27632 (Tocris, 1254). The medium was changed daily (without Y-27632) until hiPSCs reached roughly 60% confluency.

For embryoid body formation, hiPSCs were dissociated by Collagenase Type IV (Invitrogen 17104-019) and manual scraping followed by gravity sedimentation to remove as many MEFs as possible. The cells were then individualized with Accutase (Invitrogen, A1110501) and 2.4×10^6^ cells were seeded per well of an AggreWell 400 (STEMCELL Technologies, 34411) following the manufacturer's instructions by centrifugation in X-VIVO 10 medium (Lonza, 04-380Q) with 10 µM Y-27632. The following day, spheroids were extracted from the AggreWell 400 according to manufacturer's instructions and cultured in ultra-low attachment dishes (Corning, 3262) with serum-containing medium for up to 4 days with a medium change every other day.

### Endoderm-free embryoid body culture

Before endoderm-free embryoid body experiments, feeder-free hiPSCs were maintained on Matrigel in E8 medium. hiPSCs were dissociated with Accutase and 1.2×10^6^ cells were seeded per well of an AggreWell 400 by centrifugation in E8 with 10 µM Y-27632. The next day, spheroids were transferred to ultra-low attachment 6-well plates (Corning, 3471) in E8 supplemented with laminin (Invitrogen, 23017015) for 48 h. 140 µg/ml laminin was used except where otherwise stated in the main text.

### Western blotting

For hiPSCs, adherent cultures in 100 mm dishes were rinsed in ice-cold PBS and then lysed in 200 µl RIPA buffer with protease and phosphatase inhibitors. For endoderm-free embryoid bodies, suspension cultures were transferred to 1.5 ml tubes and rinsed 3 times in ice-cold PBS to remove unbound laminin, followed by lysis in 100 µl RIPA buffer with protease and phosphatase inhibitors and homogenization using 10 strokes of a sterile pestle. Samples were then centrifuged at 10,000 ***g*** for 10 min at 4°C and the supernatant soluble fraction was used for SDS-PAGE and western blotting.

For western blots on total protein, 15 µg protein per sample was denatured by 5 min incubation at 95°C in SDS-PAGE loading buffer, resolved on 4–12% Bis-Tris gels and finally transferred to PVDF membranes. For the remaining western blots, which were on the glycoprotein fraction, 100–500 μg protein was incubated overnight at 4°C in RIPA buffer (1 µg/µl) with 20–50 µl agarose-bound wheat germ agglutinin (Vector Labs, AL-1023) to pull down the glycoprotein fraction. The agarose was washed three times with RIPA, and the glycoproteins were eluted by 5 min incubation at 95°C in SDS-PAGE loading buffer. Glycoproteins were separated on 4–12% Bis-Tris gels and transferred to PVDF membranes.

All blocking steps and antibody incubations were performed in TBST with 5% milk (glyco-αDG and βDG) or 5% donkey serum (core-αDG). The membranes were probed with antibodies against glyco-αDG, core-αDG or βDG overnight at 4°C. Labeling was visualized by chemiluminescence with appropriate secondary HRP-conjugated antibodies on a ChemiDoc XRS+ (Bio-Rad). See Table S2 for details of antibodies used in this study.

### Laminin overlay assay

PVDF membranes were first blocked with 5% milk in laminin binding buffer (LBB; 140 mM NaCl, 10 mM triethanolamine, 1 mM CaCl_2_, 1 mM MgCl_2_, 0.05% Tween, pH 7.6) and then incubated with 1 µg/ml laminin in LBB overnight at 4°C. PVDF membranes were washed and probed with laminin antibodies for 1 h at room temperature in LBB with 5% milk. The membranes were then washed and probed with an appropriate HRP-conjugated secondary antibody for 1 h at room temperature in LBB with 5% milk before chemiluminescence imaging.

### Immunofluorescence microscopy

For immunocytochemistry, cells in chamber slides were fixed for 10 min in 4% paraformaldehyde (PFA) and then washed with PBS before staining. For immunohistochemistry, embryoid bodies were fixed for 20 min in 4% PFA and cryoprotected by overnight incubation with 30% sucrose in PBS. The next day, the embryoid bodies were loosely pelleted in optimum cutting temperature (OCT) compound (VWR, 25608-930) by 1 min centrifugation at 1000 ***g***. Embryoid bodies were then frozen in OCT blocks, sectioned at 10 µm thickness on a cryostat, and mounted on slides for staining.

Slides were blocked in 10% goat serum and 0.1% Triton X-100 for 1 h at room temperature before primary antibody incubation with 3% goat serum overnight at 4°C. Secondary antibody labeling was performed at room temperature for 1 h. Refer to Table S2 for antibody dilutions and catalog numbers. Fluorescent images were captured on a Leica TSC SP5 II confocal microscope or a Nikon Eclipse Ti-E inverted microscope.

### Transmission electron microscopy

Embryoid bodies were fixed for 30 min at room temperature in 0.1 M cacodylate buffer with 4% glutaraldehyde, pH 7.4. Samples were then coated in agarose, washed with buffer, and incubated for 60 min at 4°C in 0.1 M cacodylate buffer with 1% osmium tetroxide, pH 7.4. The samples were washed and stained *en bloc* overnight at 4°C in 0.1 M acetate buffer with 1% uranyl acetate, pH 5.0. The next day, samples were dehydrated in ethanol and epoxy resin embedded. 70 nm sections were cut and counterstained with lead citrate and uranyl acetate. Micrographs were captured on a JEOL1200EX transmission electron microscope with a digital CCD camera (AMT XR-100, Danvers, MA, USA).

### Image quantification and statistics

The image processing program Fiji was used to analyze all western blots and microscopy images, and the microscopy analyses were performed blind to sample genotype and treatment. All culture experiments were validated with triple antibody labeling, including at least one antibody against OCT4 or SOX17 to label the epiblast-like or endoderm-like lineages, respectively. Before capturing an image, embryoid bodies were further assessed by the polarized nuclear morphology of the epiblast-like layer seen in the DAPI staining, so that only morphologically mature embryoid bodies were considered for analysis. To measure IIH6C4 antibody labeling in western blot and immunohistochemistry, equal sized regions of interest were drawn in each sample image, and the average pixel intensity was measured. To quantify basal lamina thickness, electron micrographs were used to measure a line from the cell membrane to the outermost aspect of the basal lamina – the average of three lines were taken from each micrograph. For embryoid body size measurements, phase-contrast images were used to draw a region of interest around each embryoid body, and the area in µm^2^ was calculated. To determine laminin surface accumulation in endoderm-free embryoid bodies, immunohistochemistry images were used to measure the µm coverage of laminin fluorescence on each embryoid body, and the percentage of surface laminin was calculated for each embryoid body by dividing the laminin µm value by the embryoid body circumference. Before statistical measurements, all data were assessed for normality using the Shapiro–Wilk test. If data were not normally distributed, they were analyzed by Kruskal–Wallis test with Dunn's correction for multiple comparisons (Fig. 5E). If data were normally distributed, an ordinary one-way ANOVA was used and corrected with Tukey's multiple comparisons test (all other figures). The number of replicates, *n*, for each analysis are reported in their respective figure legends. Prism 7.0 (GraphPad software) was used to make all statistical tests and graphs. Figures were generated with Adobe Illustrator 2019.

## Supplementary Material

Supplementary information
