# Supplementary material for: Human embryoid bodies as a 3D tissue model of the extracellular matrix and α-dystroglycanopathies
Source: Dis Model Mech. 2020 Jun 26;13(6):dmm042986. doi: 10.1242/dmm.042986 (PMC7328151; doi:10.1242/dmm.042986)
Supplement: Supplementary information [file dmm-13-042986-s1.pdf]

Supplementary information

Supplementary figures

Figure S1

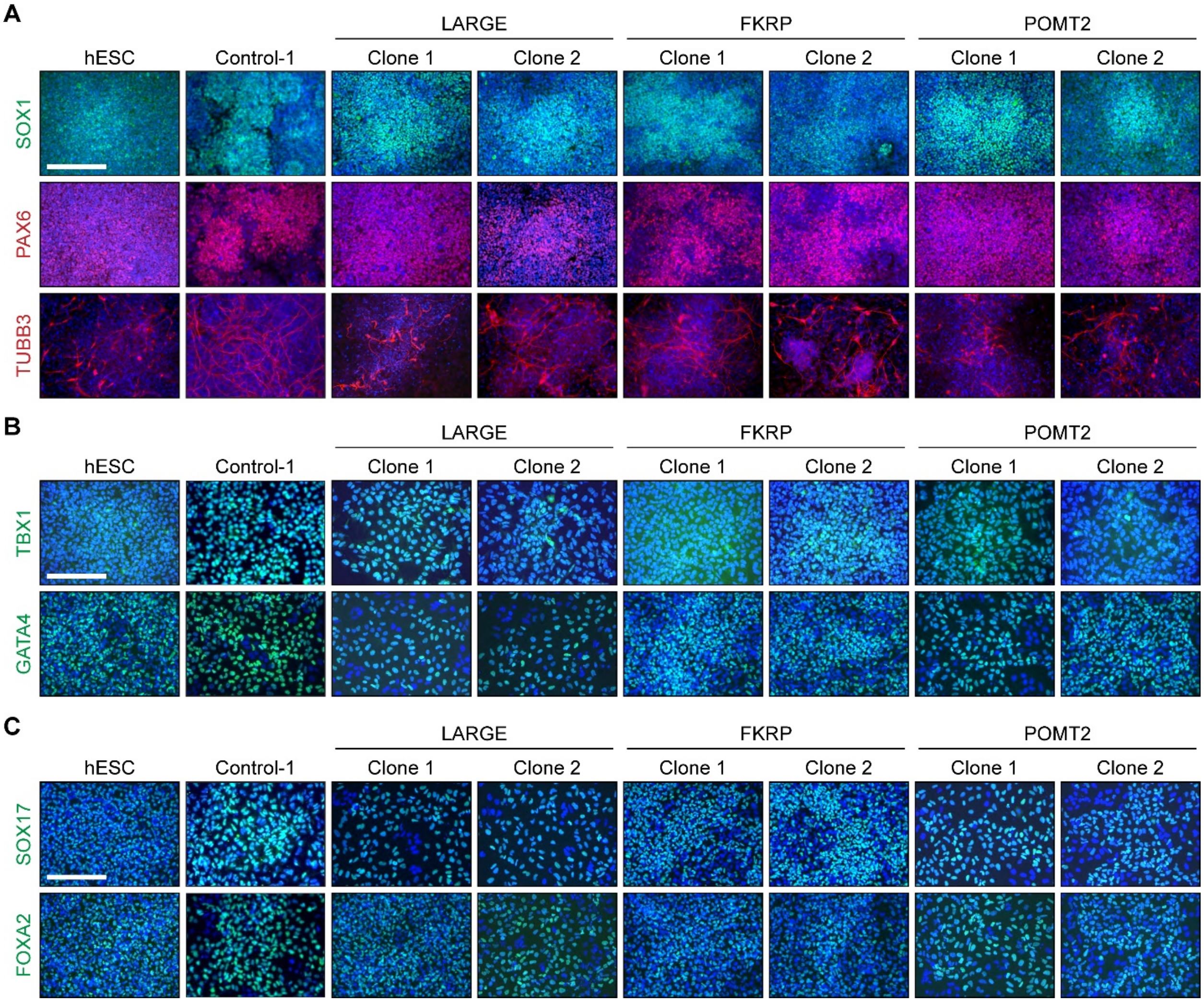

Journal of Experimental Biology • Supplementary information

Fig. S1. hiPSCs from α-dystroglycanopathy patients show normal germ layer differentiation. (A) Immunocytochemistry of ectoderm markers. hiPSCs were differentiated toward an ectoderm lineage via dual SMAD inhibition by treating the cultures with 1 μM LDN-193189 and 10 μM SB431542. Immunocytochemistry was performed after 6 days. (B, C) Immunocytochemistry of mesoderm and endoderm markers, respectively. Mesendoderm differentiation was mediated by treatment with 100 ng/mL Activin A and 75 ng/mL Wnt3a, and immunocytochemistry was performed after 3 days. hESC, H9 human embryonic stem cell line. Scale bars, 100 μm.

Figure S2

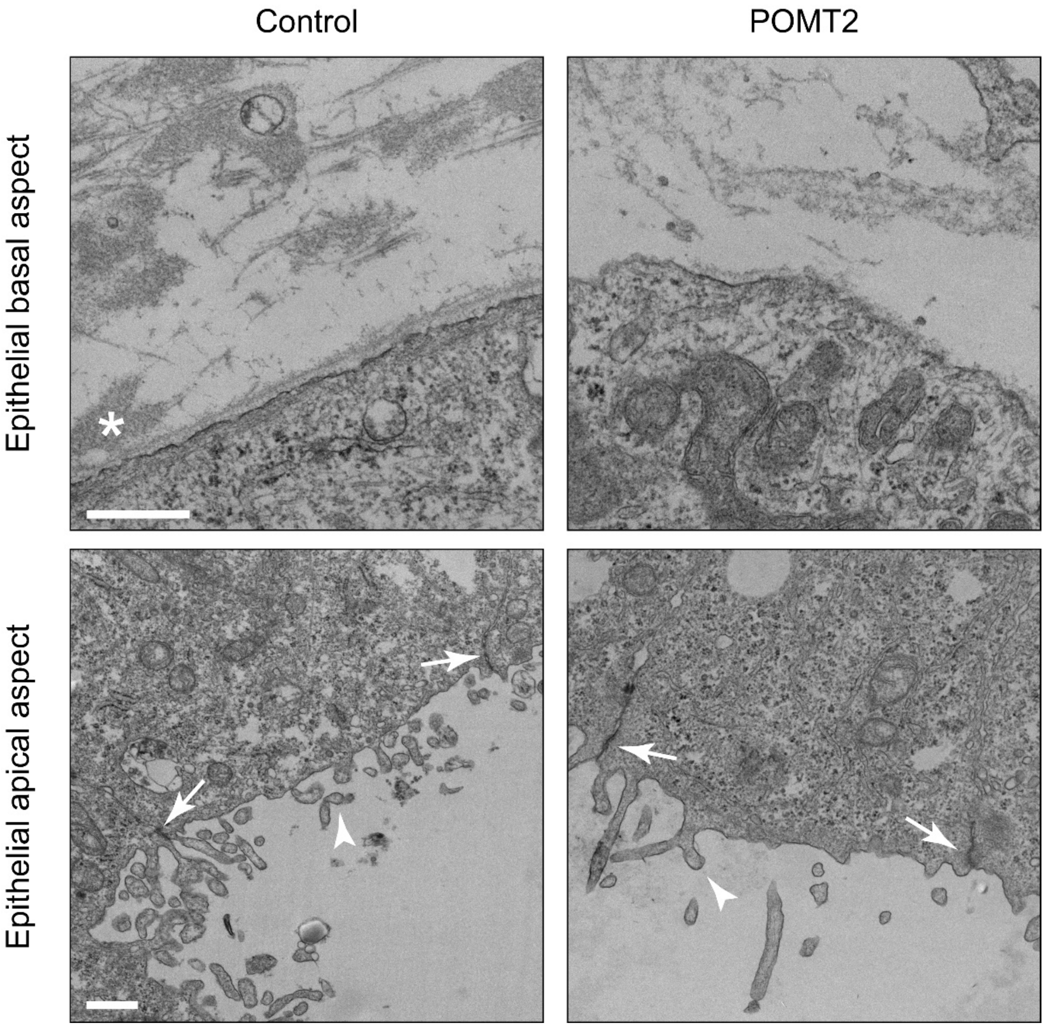

Fig. S2. Ultrastructural examination of embryoid body morphology. Transmission electron micrographs depicting the ultrastructural morphology of embryoid body epithelium. Rare attachment of fibrillar matrix to the basal lamina indicated by asterisk. Arrows demonstrate epithelial tight junctions, and the arrow head indicates apical microvilli. Scale bar, 500 nm.

Journal of Experimental Biology • Supplementary information

Figure S3

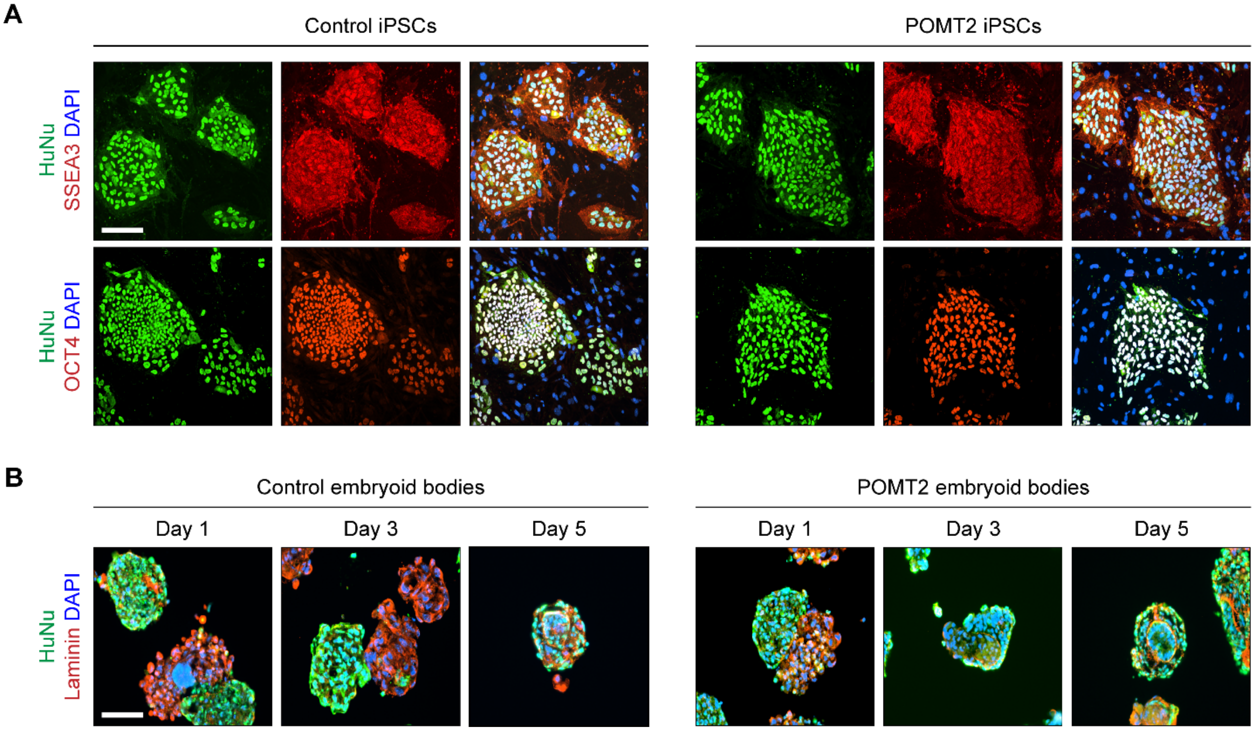

Fig. S3. Morphologically mature embryoid bodies are virtually devoid of MEFs. (A) Immunocytochemistry of feeder-dependent hiPSCs to distinguish human cells (HuNu) from MEFs. Control-1 hiPSCs and one hiPSC clone of the POMT2 patient were used. (B) Embryoid bodies at different time points, derived from control and POMT1 hiPSCs. Scale bars, 100 µm.

Journal of Experimental Biology • Supplementary information

Figure S4

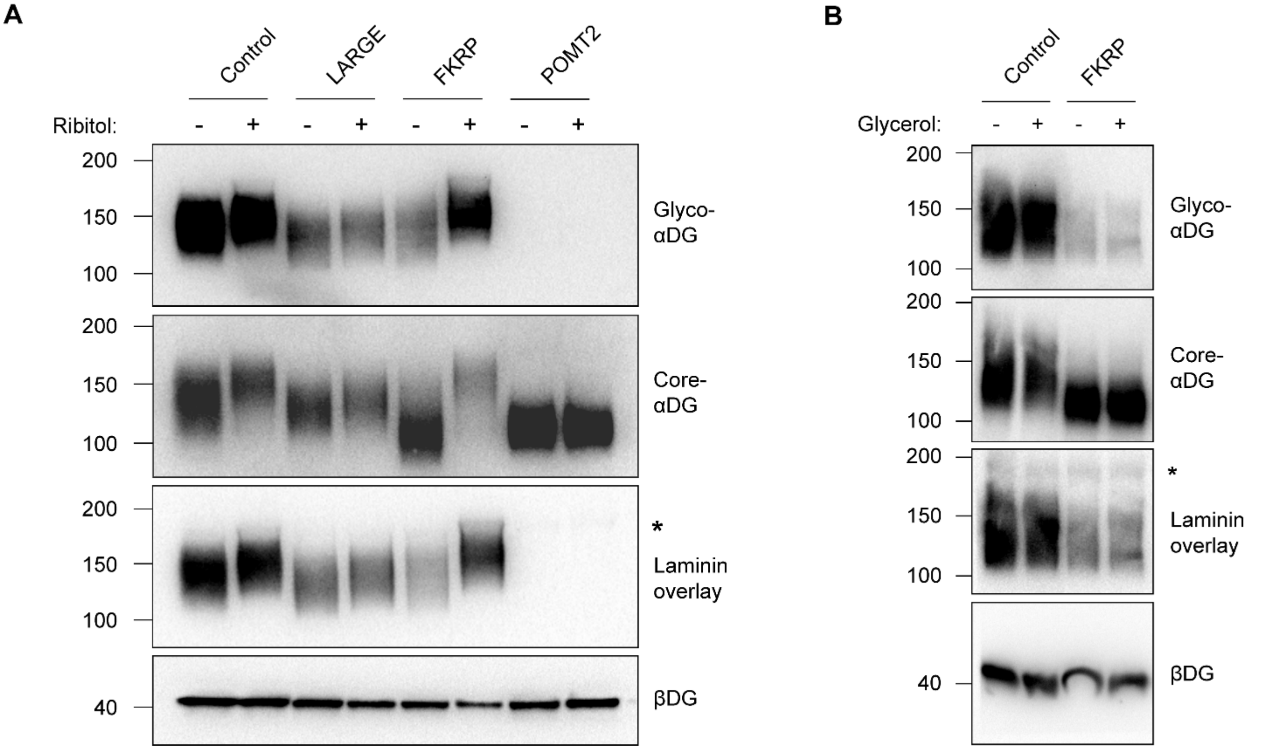

Fig. S4. Ribitol treatment promotes functional glycosylation of αDG in FKRP hiPSCs. (A) Western blotting of protein lysates from hiPSC cultures. hiPSCs were supplemented with daily medium changes with (+) or without (-) 3 mM ribitol for 72 hours before protein was collected. Asterisk indicates the position of endogenous laminin. (B) Daily administration of 3 mM glycerol for 72 hours shows no effect on αDG by western blot.

Journal of Experimental Biology • Supplementary information

Supplementary tables

Table S1

Characteristics of study subjects

Subject	Age	Gender	Clinical Presentation	Diagnosis	Genotype
**Control-1**	Neonate	Male	N/A	N/A	N/A
**Control-2**	Neonate	Male	N/A	N/A	N/A
**Control-3**	51	Female	N/A	N/A	N/A
**LARGE**	6	Female	Delayed motor milestones, weakness and general hypotonia, able to walk stairs, mild autistic behavior, mild muscle biopsy, lissencephaly on brain MRI	Congenital muscular dystrophy type 1D	LARGE1: Heterozygous - Exon 7 deletion - Exons 3 – 7 deletion
**FKRP**	3	Male	Delayed motor milestones, mild weakness, trunk hypotonia, waddling gait, able to run, mild autistic behavior, moderate dystrophy on muscle biopsy, normal basement membrane on TEM	Limb-girdle muscular dystrophy type 2I	FKRP: Heterozygous - C826A (p.L276I) - G534T (p.W178C)
**POMT2**	4 ½	Female	Severely delayed motor milestones, hip dysplasia at birth, weakness and general hypotonia, unable to stand, joint contractures, failure to thrive, delayed speech acquisition, white matter hyperintensities on MRI	Muscle-Eye-Brain Disease	POMT2: Homozygous - G1057A (p.G353S)

Age in years; TEM, transmission electron microscopy; MRI, magnetic resonance imaging

Journal of Experimental Biology • Supplementary information

Table S2

Antibodies for immunofluorescence and western blots

Antibody	Dilution	Company	Catalog Number
**SOX17**	1:100	Abcam	ab84990
**OCT4**	1:200	Abcam	ab134218
**Laminin**	1:1,000 (IF)<br>1:5,000 (WB)	Sigma-Aldrich	L9393
**Glyco-αDG**<br>(IIH6C4)	1:200 (IF)<br>1:1,000 (WB)	Millipore	05-593
**Core-αDG**	1:500	R&D Systems	AF6868
**βDG**	1:10,000	GeneTex	GTX124225
**Nestin**	1:500	Millipore	ABD69
**SSEA3**	1:200	Abcam	ab16286
**SSEA4**	1:500	STEMCELL Technologies	60062
**F-actin**<br>(Phalloidin-647)	1:100	Thermo Fisher	A22287
**Perlecan**	1:100	Millipore	MAB1948-P
**Nidogen**	1:50	R&D Systems	MAB2570
**COLIV**	1:1,000	Chemicon	MAB1430
**HuNu**	1:100	Millipore	MAB1281

IF, immunofluorescence; WB, western blot

Journal of Experimental Biology • Supplementary information
